# Non-invasive tests for liver fibrosis assessment in patients with chronic liver diseases: a prospective study

**DOI:** 10.1038/s41598-022-08955-x

**Published:** 2022-03-22

**Authors:** Kessarin Thanapirom, Sirinporn Suksawatamnuay, Natthaporn Tanpowpong, Bundit Chaopathomkul, Supachaya Sriphoosanaphan, Panarat Thaimai, Nunthiya Srisoonthorn, Sombat Treeprasertsuk, Piyawat Komolmit

**Affiliations:** 1 Division of Gastroenterology, Department of Medicine, Faculty of Medicine, Chulalongkorn University and King Chulalongkorn Memorial Hospital, Thai Red Cross Society, Bangkok, Thailand; 2 Center of Excellence in Liver Diseases, King Chulalongkorn Memorial Hospital, Thai Red Cross Society, Bangkok, Thailand; 3 Research Unit of Liver Fibrosis and Cirrhosis, Chulalongkorn University, Bangkok, Thailand; 4 Department of Radiology, Faculty of Medicine, Chulalongkorn University and King Chulalongkorn Memorial Hospital, Bangkok, Thailand

**Keywords:** Diseases, Gastroenterology, Medical research

## Abstract

There is an urgent need of non-invasive tests (NITs) for monitoring treatment response and disease progression in chronic liver disease. Liver stiffness (LS) evaluated by transient elastography (TE), shear wave elastography (SWE), and magnetic resonance elastography (MRE) and serum markers e.g. APRI and FIB-4 scores were assessed at baseline and the 1-year follow-up. In all, 89 chronic hepatitis C virus (HCV) patients with sustained virological response and 93 non-alcoholic fatty liver disease (NAFLD) patients were included. There was a significantly strong correlation among imaging techniques. Using MRE as the reference standard, the area under the receiver operating characteristics curves for TE, SWE, APRI, and FIB-4 in detecting stage1–4 fibrosis were 0.88–0.95, 0.87–0.96, 0.83–0.89, and 0.79–0.92, respectively. In chronic HCV patients, the values of TE, SWE, MRE, APRI and FIB-4 significantly decreased from baseline to the 1-year follow-up. Liver steatosis did not significantly change over time. In NAFLD, compared to obese patients, non-obese patients had less LS and steatosis at baseline, and these values did not show significant changes at the 1-year follow-up. Our study suggests that the current NITs have a good correlation and accuracy in monitoring the treatment outcomes in patients with chronic liver diseases.

## Introduction

Assessment of liver fibrosis has been a cornerstone of evaluating patients with any chronic liver disease (CLD) for therapeutic decision-making, prognostication, and evaluation of treatment response. Although liver biopsy is currently the reference standard for the identification and staging of liver fibrosis, its role has significantly declined because of several limitations such as invasiveness of the procedure, sampling errors, and intra- and interobserver variability^1,2^. The advent of non-invasive tests (NITs) has now replaced the role of liver biopsy. These approaches can overcome the limitations of liver biopsy and have become more widely used in routine clinical practice. Imaging-based elastography and serum biomarkers are currently the two main NITs for liver fibrosis staging. Liver stiffness (LS) measurement by imaging techniques such as transient elastography (TE), shear wave ultrasound elastography (SWE), and magnetic resonance elastography (MRE) have been investigated for their diagnostic performance in fibrosis staging and prediction of clinical outcomes in various CLDs^3–5^. Simple biomarker blood tests for fibrosis, including the aspartate aminotransferase-platelet ratio index (APRI) and fibrosis-4 index (FIB-4) score have been extensively validated and used given their easy accessibility^6^. In terms of non-invasive quantification of liver steatosis, TE-based controlled attenuation parameter (CAP) and magnetic resonance imaging-estimated proton density fat fraction (MRI-PDFF) have been investigated in patients with several CLDs, particularly non-alcoholic fatty liver disease (NAFLD)^3,7,8^. The accuracy of CAP and MRI-PDFF for diagnosing all grades of steatosis in patients with NAFLD was 0.85 and 0.99, respectively^3^. MRI-PDFF has been reported to perform better than CAP for detecting liver steatosis^3,7^.

Several issues need to be addressed regarding the aetiology-specific consideration of NITs for staging of liver fibrosis. In chronic hepatitis C virus (HCV) infection, the introduction of direct-acting antiviral (DAA) therapies has led to a paradigm shift in the treatment eradication results in high sustained virological response (SVR) achievement. Previous studies have shown that serum markers and TE values decline following SVR in chronic HCV-infected patients treated with pegylated-interferon-based regimens, and this is likely to be influenced by the early reduction of liver necro-inflammation^9–12^. The role of LS measurement after SVR is an essential clinical concern in the current DAA era. Currently, data on the effect of SVR following DAA therapy for liver fibrosis evaluated by imaging-based techniques and simple serum biomarkers are scarce. Another issue is in patients with non-obese NAFLD, defined as body mass index < 25 kg/m^2^, who appear to be recognized more common in Asians^13,14^. The clinical characteristics, prognosis, severity, and liver fibrosis progression in these patients are not well understood. The data on LS values evaluated by imaging tests and simple serum biomarkers and the changes of these parameters in non-obese NAFLD patients compared to those with obese NAFLD patients have previously not been well investigated.

There is a clinical need to determine whether LS and liver steatosis values obtained by using different NITs are indeed interchangeable. Therefore, this study aimed to investigate the correlation of LS and steatosis values among imaging tests and simple serum biomarkers. Moreover, the diagnostic performance of TE, SWE, APRI, and FIB-4 score by using MRE as the reference standard was evaluated. In addition, we prospectively assessed the changes of serum simple fibrosis marker; LS values evaluated by TE, SWE, and MRE; and liver steatosis in chronic HCV patients who achieved SVR after DAA therapy and NAFLD patients at baseline and 1-year follow-up.

## Methods

### Study participants

**Figure 1:**
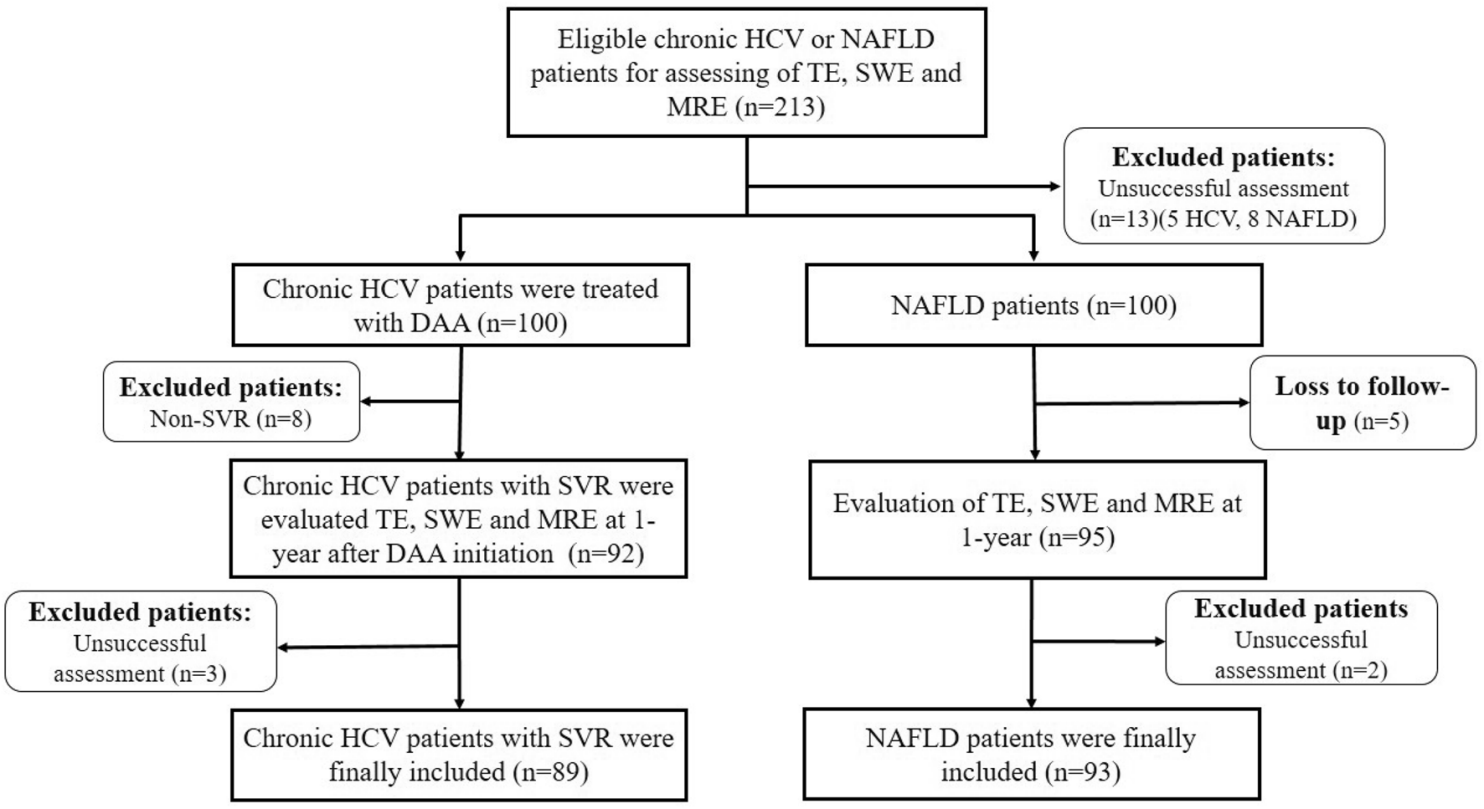
Flow chart of patient enrollment.

This prospective observational cohort study was conducted at the Hepatology Clinic, King Chulalongkorn Memorial Hospital, Chulalongkorn University, Bangkok, Thailand. Patients aged ≥ 18 years with chronic HCV infection or NAFLD were enrolled between July 2017 and June 2018. The inclusion criteria were: (1) patients with chronic HCV infection who received a 12- or 24-week course of DAA therapy and achieved SVR at 12 weeks after treatment completion; or (2) patients in whom NAFLD was diagnosed based on ultrasonography findings of fatty liver without other causes of CLD. The exclusion criteria were: (1) presence of other causes of CLD such as hepatitis B virus infection, alcohol consumption > 80 g/day, autoimmune liver diseases, hemochromatosis, and Wilson’s disease; co-infection with human immunodeficiency virus (HIV); (2) chronic HCV patients without SVR after DAA treatment; (3) unsuccessful assessment of LS and liver steatosis using Fibroscan, ultrasound, and MRI at baseline and 1-year follow-up, and (4) patients with some confounders that influence the reliability of liver elastography, including biliary obstruction, decompensated heart failure, and pregnant women. The flow chart of patient enrolment is shown in Fig. 1.

The study protocol and patient consent form were approved by the Institutional Review Board, Faculty of Medicine, Chulalongkorn University (IRB 424/59). The study protocol was in compliance with the ethical principles of the Helsinki Declaration and followed the Good Clinical Practice guidelines. All participants provided written informed consent to participate in the study. The study protocol was registered at the Thai Clinical Trial Registry (TCTR20210430002, First submitted date; 23rd April 2021).

### Assessment of liver stiffness and steatosis

All studied participants underwent LS measurement using three imaging elastography techniques, namely TE, SWE, and MRE, before DAA therapy and at 1-year after treatment initiation. All elastography tests were done on the same day.

TE was performed using a Fibroscan® 520 Touch (Echosens, Paris, France) on the right liver lobe by a 3.5 MHz M or 2.5 MHz XL probe, according to the manufacturer’s instructions. The M probe was used initially, followed by the XL probe if the M probe failed. Patients were investigated under at least 2 hours of fasting. The measurement depth was between 25 and 65 mm. The TE result was considered valid if the following criteria were met; (1) ≥ 10 successful measurements; (2) a success rate ≥ 60%; and (3) an interquartile range (IQR)/median ratio < 30%^15^. Meanwhile, liver steatosis was evaluated using the CAP value provided by the machine and expressed in decibel/milliwatt (dB/m). The mean and IQR value of successful LS and CAP measurements was calculated by the device. One official certified (PT) operator was responsible for LS and CAP measurement.

Two-dimensional SWE was performed using a LOGIQ E9 (GE Healthcare, Milwaukee, WI, USA) and a convex probe (1–6 MHz) by one of two abdominal radiologists (BC and NT), both of whom had evaluated the SWE in more than 100 patients. The operators were blinded to the results obtained by other techniques during the measurement. The right liver lobe was visualized through an intercostal space, and a region of interest (ROI) was placed at less than 5 cm below the liver surface and avoided major vessels. Measurements were taken 10–15 times while the patients held their breath for 5–10 seconds. The result was considered reliable when 10 successful shots and an IQR/median ratio < 30% was required. Finally, the LS value was automatically calculated as kilopascals (kPa) using shear wave velocity in the liver tissue by the machine.

MRE was performed using the Discovery™ 750w 3.0 T MRI system (GE Healthcare, Milwaukee, WI, USA) according to the protocol described previously^16^. In brief, a passive driver was placed on a supine patient over the right upper quadrant of the abdominal wall. The driver generated low-amplitude 60-Hz vibrations into the liver and the signal can be measured in the two-dimensional gradient echo sequences (2D GRE). MRE pulse sequence was used to capture the propagation of shear wave. LS values were obtained by drawing ROIs around the whole liver on each axial slice, avoiding the edges of the liver and large blood vessels. The ROI was placed by one of two abdominal radiologists (BC and NT) with more than 5 years’ clinical experience. Mean LS (kPa) was calculated from three individual slices. Patients were instructed to hold their breath while obtaining each slice. The LS was considered reliable if the coefficient of variation (CV) was < 30%. Liver steatosis was evaluated using MRI-PDFF with a validated multi-echo gradient-echo pulse sequence^17^. The PDFF map was estimated as the ratio of fat signal over the sum of fat and water signals. Based on a previous study, LS cut-off values by MRE for mild fibrosis (≥ F1), significant fibrosis (≥ F2), advanced fibrosis (≥ F3), and cirrhosis (F4) were 2.3, 3.2, 4, and 4.6 kPa, respectively^18^.

### Direct-acting antiviral treatment

Regimens and duration of DAA therapy were determined based on the 2018 Thailand Practice Guideline for the Management of Chronic Hepatitis C^19^. Patients with chronic HCV infection were treated with sofosbuvir (400 mg)/daclatasvir (60 mg) or sofosbuvir (400 mg)/ledipasvir (90 mg) with or without ribavirin 600–1200 mg each day for 12–24 weeks.

### Clinical and laboratory parameters

The baseline characteristics of all patients were recorded. Serum aspartate aminotransferase (AST), alanine aminotransferase (ALT), total bilirubin (TB), albumin, white blood cell (WBC) count, platelet count, and prothrombin time; serum lipid profile; and fasting plasma glucose were quantified in all patients at baseline and at the 1-year follow-up. These laboratory tests were additionally performed at SVR12 in patients with chronic HCV infection. Simple serum biomarkers for liver fibrosis including APRI and FIB-4 index were determined at baseline and the 1-year follow-up. The APRI score was calculated using the formula: [AST (U/L)/AST (upper limit of normal (U/L))]/platelet count (10^9^/L) × 100. The FIB-4 index was calculated using the following equation: [Age (years) × AST (U/L)]/[platelet count (10^9^/L) × √AST (U/L)]^20,21^.

In chronic HCV patients, HCV RNA quantification was performed before and at SVR12 using a real-time reverse transcription-polymerase chain reaction (PCR)-based method (COBAS Taqman HCV assay, Roche Diagnostics, Basel, Switzerland). HCV genotype was assessed via the INNO-LiPA HCV II assay (Innogenetics, Ghent, Belgium). SVR was confirmed as undetectable HCV RNA in the plasma 12 weeks after the completion of treatment (SVR12). Liver cirrhosis was diagnosed by radiological imaging.

### Statistical analysis

Continuous variables were expressed as median and standard deviation. The Mann–Whitney *U* test was used to compare continuous variables, and Pearson’s chi-square or Fisher’s exact test was used for categorical variables. Correlations between the value of serum fibrosis score, LS, and liver steatosis values were assessed using Spearman’s correlation coefficient. For all analyses, P < 0.05 was considered to indicate statistical significance. The diagnostic accuracy of the studied NITs was evaluated by calculation of the area under the receiver operating characteristic (AUROC) curves using MRE as the reference. Statistical analysis was performed using SPSS software (version 22, IBM Corporation, Armonk, NY, USA).

## Results

### Patient baseline characteristics

A total of 182 patients were finally enrolled during the study period. Of these, there were 89 patients with chronic HCV infection and 93 with NAFLD. The patients consisted of 97 (53.3%) females with a mean age (standard deviation: SD) of 53.5 (12.4) years. Forty-five patients (24.7%) had liver cirrhosis. Baseline characteristics, demographic data, laboratory parameters, LS, and steatosis values are shown in Table 1.

**Table 1:** Patient baseline characteristics.

	Overall (n = 182)	Chronic HCV (n = 89)	NAFLD (n = 93)
Age (years)	53.5 ± 12.4	57.3 ± 9.7	49.6 ± 13.7
Female, n (%)	97 (53.3%)	46 (51.7%)	51 (54.8%)
Diabetes mellitus, n (%)	30 (17.1%)	15 (17.4%)	15 (16.1%)
Body mass index, kg/m^2^	26.2 ± 5.0	24.6 ± 3.9	27.7 ± 5.4
Waist circumference (inches)	35.3 ± 7.2	34.2 ± 7.5	36.3 ± 6.9
Cirrhosis, n (%)	45 (24.7%)	43 (48.3%)	2 (2.2%)
Hemoglobin (g/dL)	13.6 ± 1.7	13.4 ± 1.7	13.9 ± 1.5
White cell count (× 10^9^/L)	6.2 ± 1.9	5.4 ± 1.7	7.1 ± 1.7
Platelet count (× 10^9^/L)	220.6 ± 95.2	175.1 ± 95.3	273.1 ± 62.8
Total bilirubin (mg/dL)	1.0 ± 0.8	1.1 ± 0.9	0.7 ± 0.4
AST (IU/L)	52.1 ± 41.2	72.7 ± 46.7	31.6 ± 19.6
ALT (IU/L)	65.9 ± 59.8	82.5 ± 69.0	49.0 ± 43.0
Albumin (g/dL)	4.0 ± 0.4	3.8 ± 0.4	4.3 ± 0.3
Creatinine (mg/dL)	0.9 ± 0.3	0.9 ± 0.3	0.8 ± 0.2
APRI score	1.0 ± 1.4	1.5 ± 1.7	0.3 ± 0.2
FIB-4 index	2.4 ± 2.7	3.7 ± 3.1	1.0 ± 0.6
TE (kPa)	12.3 ± 11.7	6.8 ± 3.4	18.0 ± 14.2
SWE (kPa)	8.2 ± 3.6	5.9 ± 1.9	10.5 ± 3.5
MRE (kPa)	3.3 ± 1.7	2.3 ± 0.5	4.3 ± 1.8
TE-based CAP (dB/m)	252.8 ± 62.2	283.5 ± 57.4	220.9 ± 50.0
MRI-PDFF (%)	8.1 ± 7.2	11.3 ± 8.4	4.9 ± 3.6

*AST* aspartate aminotransferase, *ALT* alanine aminotransferase, *APRI* AST to Platelet Ratio, *CAP* controlled attenuation parameter, *FIB-4* Fibrosis-4, *kPa* kilopascal, *MRE* magnetic resonance elastography, *MRI* magnetic resonance imaging, *PDFF* proton density fat fraction, *SWE* shear wave elastography, *TE* transient elastography.

### Correlations of liver stiffness and steatosis among imaging and serum biomarker tests

The correlation between all NITs in study participants at baseline was investigated. MRE showed a strong positive correlation with TE (r = 0.82, p < 0.001); SWE (r = 0.83, p < 0.001); and APRI score (r = 0.71, p < 0.001) and moderate positive correlation with FIB-4 score (r = 0.69, p < 0.001). SWE was strongly positively correlated with TE (r = 0.80, p < 0.001); APRI score (r = 0.72, p < 0.001); and FIB-4 score (r = 0.71, p < 0.001). TE showed a strong positive correlation with APRI score (r = 0.74, p < 0.001) and moderate positive correlation with FIB-4 index (r = 0.64, p < 0.001). Comparing the serum fibrosis scores, APRI showed strong positive correlations with FIB-4 (r = 0.88, p < 0.001). The correlation between APRI and FIB-4 was strong in patients with cirrhosis (r = 0.90, p < 0.001) and non-cirrhosis (r = 0.79, p < 0.001).

**Figure 2:**
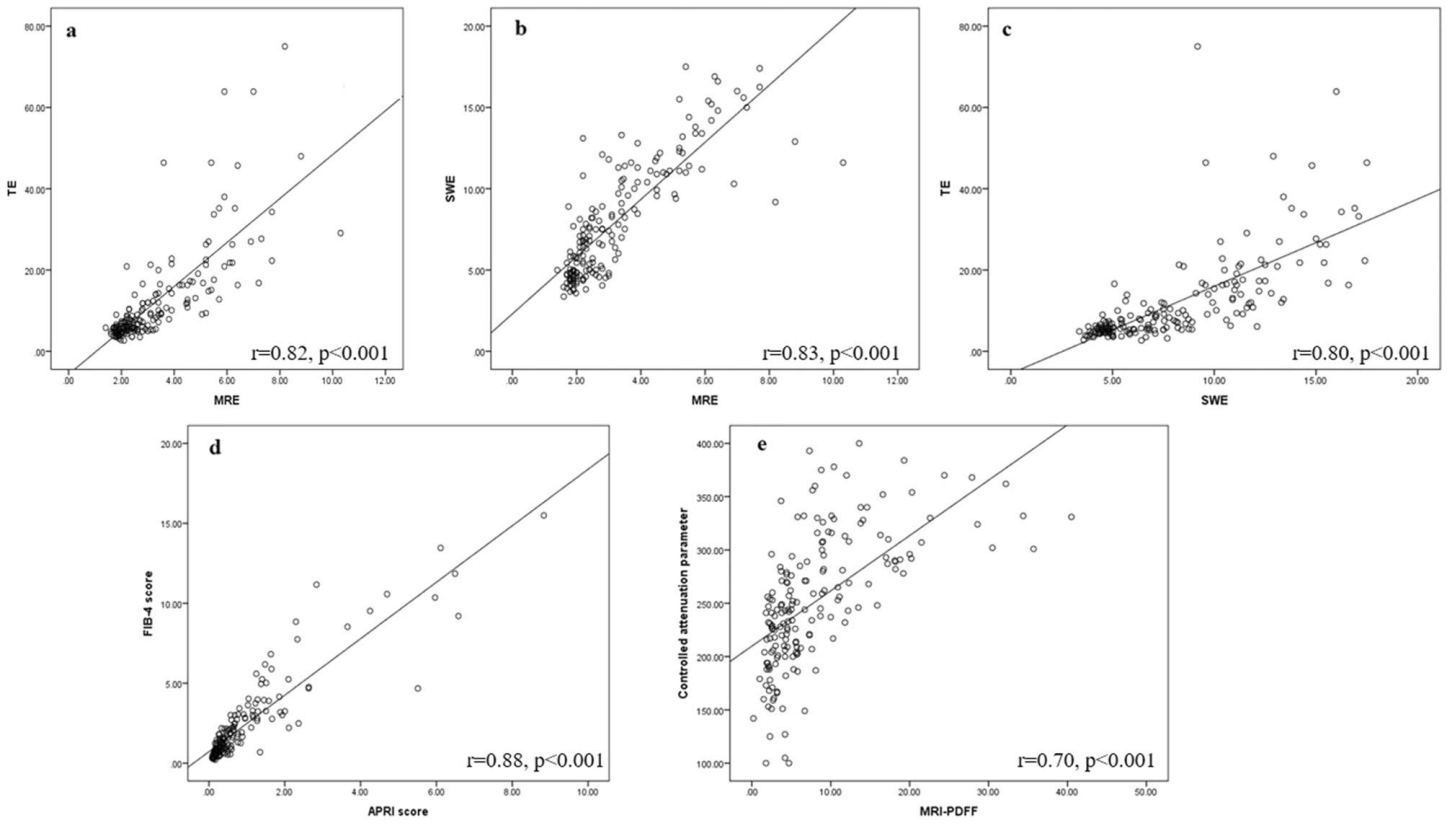
Correlation fibrosis markers and liver steatosis among non-invasive tests at baseline: (**a**) Magnetic resonance elastography (MRE) vs. Transient elastography (TE) (**b**) MRE vs. Shear wave elastography (SWE) (**c**) SWE vs. TE (**d**) APRI vs. FIB-4 score (**e**) TE-based controlled attenuated parameter vs. Magnetic resonance imaging-proton density fat fraction.

With respect to NITs for liver steatosis measurement, MRI-PDFF was strongly positively correlated with TE-based CAP (r = 0.70, p < 0.001). However, in the subgroup analysis, the strong correlation was found only in patients with non-cirrhosis (r = 0.73, p < 0.001), while there was low correlation in patients with cirrhosis (r = 0.35, p = 0.02). Figure 2 shows the correlation between the studied NITs for liver fibrosis and steatosis.

### Diagnostic performance of fibrotic markers

**Figure 3:**
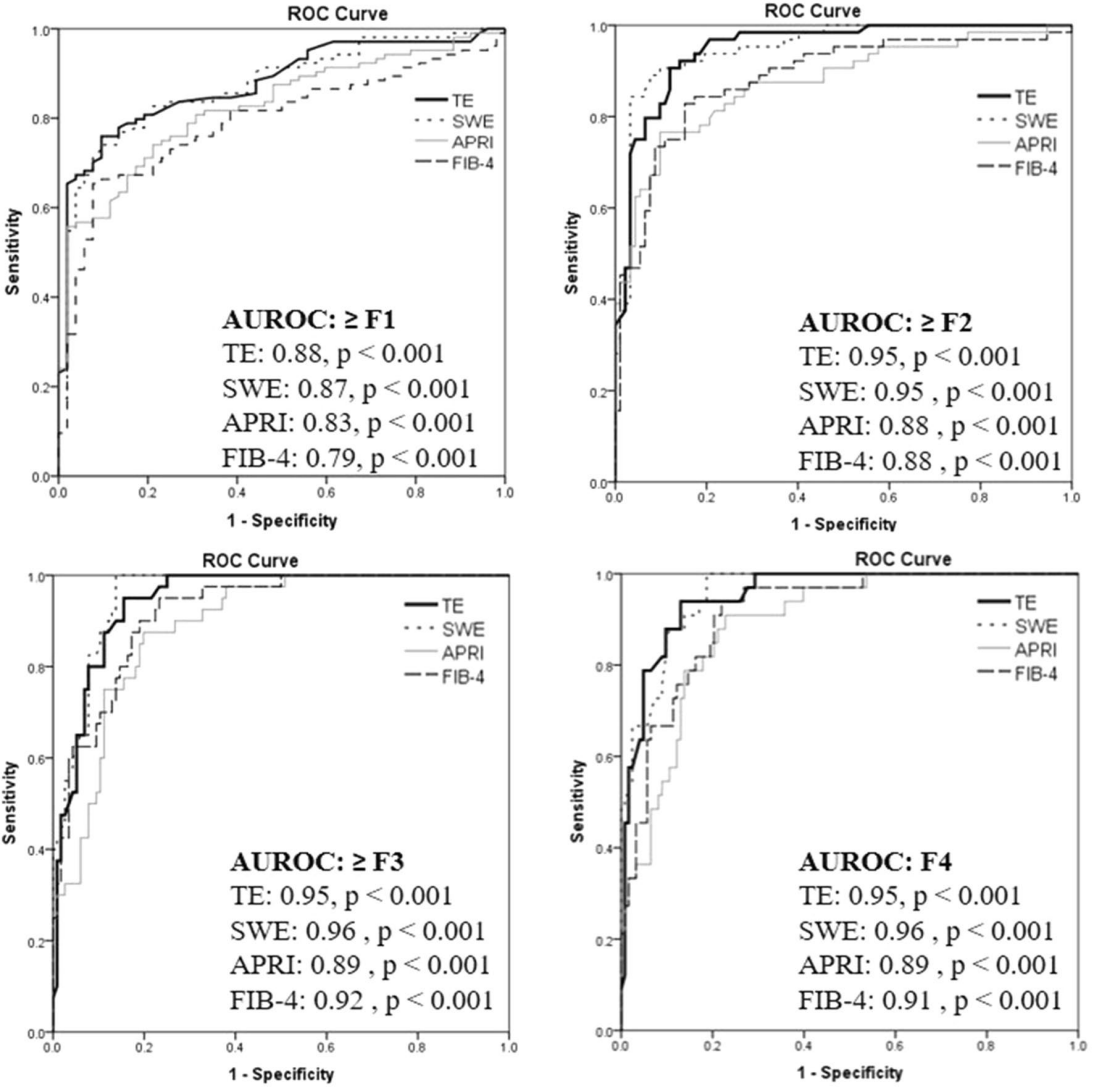
Diagnostic performance of TE, SWE, APRI and FIB-4 score using MRE as reference method.

Using MRE as the reference method for staging liver fibrosis, the diagnostic performance of TE, SWE, APRI, and FIB-4 score were calculated based on the AUROCs (Fig. 3). The AUROCs for TE in differentiating mild fibrosis (≥ F1), significant fibrosis (≥ F2), advanced fibrosis (≥ F3), and cirrhosis (F4) were 0.88 (95% CI 0.82–0.93, p < 0.001), 0.95 (95% CI 0.92–0.98, p < 0.001); 0.95 (95% CI 0.91–0.98, p < 0.001); and 0.95 (95% CI 0.92–0.99, p < 0.001), respectively. For SWE, the corresponding values were 0.87 (95% CI 0.82–0.93); 0.95 (95% CI 0.92–0.98, p < 0.001); 0.96 (95% CI 0.93–0.98, p < 0.001); and 0.96 (95% CI 0.92–0.99, p < 0.001), respectively. The corresponding figures for APRI scores were 0.83 (95% CI 0.76–0.89); 0.88 (95% CI 0.92–0.98, p < 0.001); 0.89 (95% CI 0.84–0.96, p < 0.001); and 0.89 (95% CI 0.84–0.94, p < 0.001), respectively. For the FIB-4 index, the corresponding values were 0.79 (95% CI 0.72–0.83); 0.88 (95% CI 0.83–0.94, p < 0.001); 0.92 (95% CI 0.88–0.96, p < 0.001); and 0.91 (95% CI 0.87–0.96, p < 0.001), respectively.

The distribution of LS values in kPa by TE and SWE in comparison with different fibrosis stages using MRE are shown in Supplementary Fig. 1. There were considerable overlaps in the TE and SWE readings, especially between F2 and F3. The mean (SD) values of LS using imaging tests and serum simple fibrotic score are summarized in Supplementary Table 1. The average LS values, APRI, and FIB-4 index increased with higher liver fibrosis stage.

### Simple serum fibrotic markers, liver stiffness, and steatosis in chronic HCV patients with SVR after DAA therapy

A total of 89 chronic HCV patients who achieved SVR12 after DAA therapy were enrolled. Of these, 41 patients (46.0%) were infected with HCV genotype 1, 40 patients (44.9%) with genotype 3, and eight patients (9%) with genotype 6. The number of patients who received sofosbuvir/daclatasvir with or without ribavirin and sofosbuvir/ledipasvir with or without ribavirin was 74 (83.1%) and 15 (16.9%), respectively. Pre-treatment HCV RNA was 6.1 ± 0.9 log IU/mL. Laboratory parameters and serum non-invasive fibrotic markers were evaluated before treatment, at SVR12, and one year after treatment initiation (post-SVR12). Levels of TB and serum AST and ALT significantly decreased at SVR12 (p ≤ 0.05) and post-SVR12 (p ≤ 0.05) when compared to baseline (Table 2). Serum albumin level significantly increased between baseline and post-SVR12 (p < 0.05), while the platelet and white blood cell counts did not significantly change over time. APRI and FIB-4 index were lower when compared between baseline and SVR12 (p ≤ 0.001) and between baseline and post-SVR12 (p ≤ 0.001) (Table 2).

**Table 2:** Laboratory paraemeters and serum fibrotic scores in patients with chronic HCV treated with direct-acting antivirals during SVR 12 and post-SVR12 (n = 89).

	Baseline	SVR12	Post-SVR12
Hemoglobin (g/dL)	13.4 ± 1.7	13.4 ± 1.8	13.7 ± 3.7
White cell count (× 10^9^/L)	5.4 ± 1.7	5.4 (4.3–6.8)	5.4 ± 1.9
Platelet count (× 10^9^/L)	175.1 ± 95.3	184.7 ± 79.3	172.0 ± 72.2
Total bilirubin (mg/dL)	1.1 ± 0.9	1.0 ± 0.6^#^	0.9 ± 0.6 ***
AST (IU/L)	72.7 ± 46.7	27.9 ± 11.5^###^	31.8 ± 26.4***^,@^
ALT (IU/L)	82.5 ± 69.0	24.6 ± 18.6^###^	28.3 ± 33.8***^,@^
Albumin (g/dL)	3.8 ± 0.4	4.1 ± 0.4^###^	4.1 ± 0.4***
APRI score	1.5 ± 1.7	0.6 ± 0.5^###^	0.7 ± 0.6***
FIB-4 index	3.7 ± 3.1	2.6 ± 2.1^###^	2.8 ± 2.0 ***

Baseline vs. SVR12: ^#^p ≤ 0.05, ^##^p ≤ 0.01, ^###^p ≤ 0.001; Baseline vs. post-SVR12; *p ≤ 0.05, **p ≤ 0.01, ***p ≤ 0.001; SVR12 vs. post-SVR12: ^@^p ≤ 0.05.

*AST* aspartate aminotransferase, *ALT* alanine aminotransferase, *APRI* AST to Platelet Ratio, *SVR12* sustained virological response after 12 weeks post-treatment.

**Figure 4:**
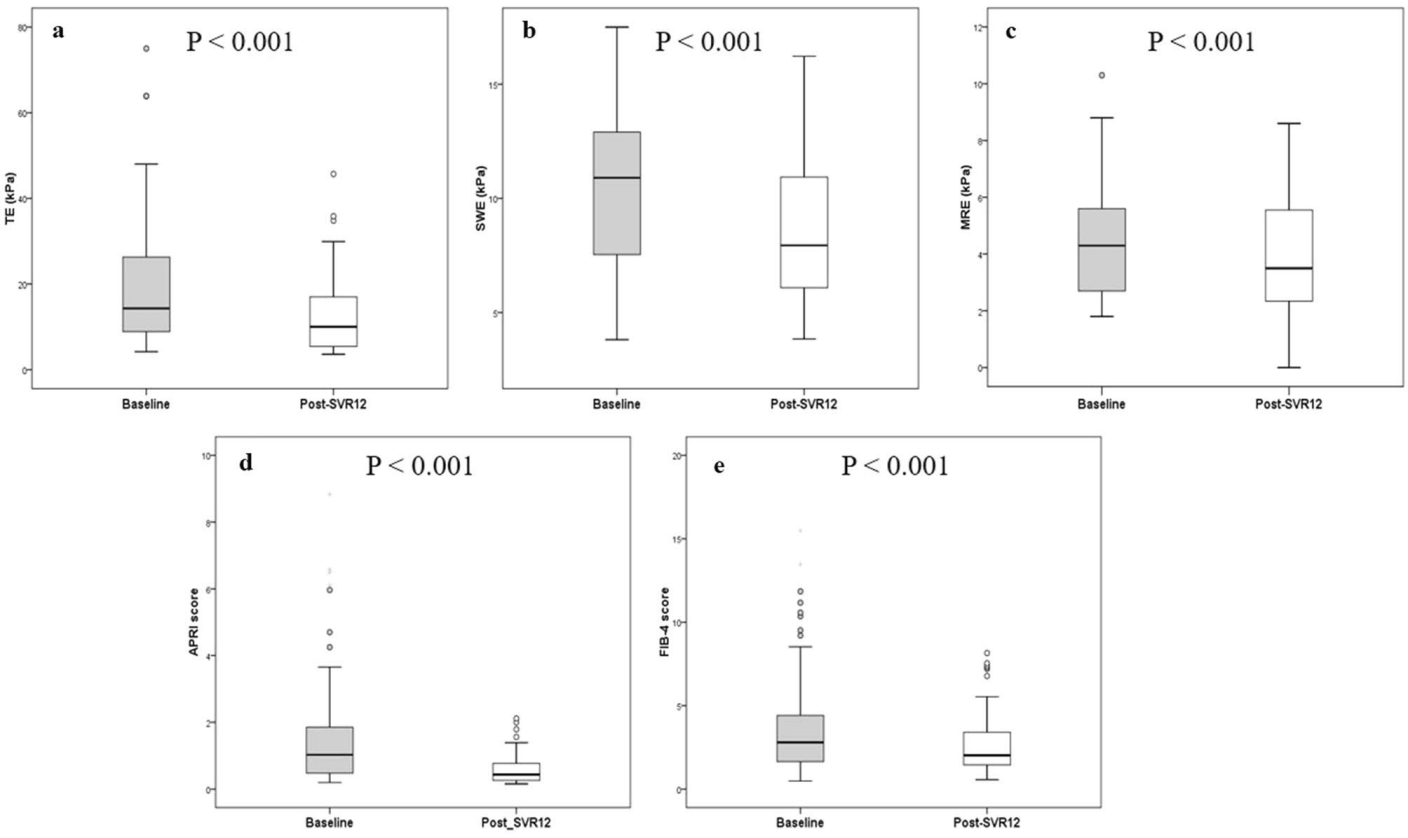
Liver stiffness values, APRI and FIB-4 scores at baseline and post-SVR 12 in chronic HCV patients with SVR after DAA treatment.

**Figure 5:**
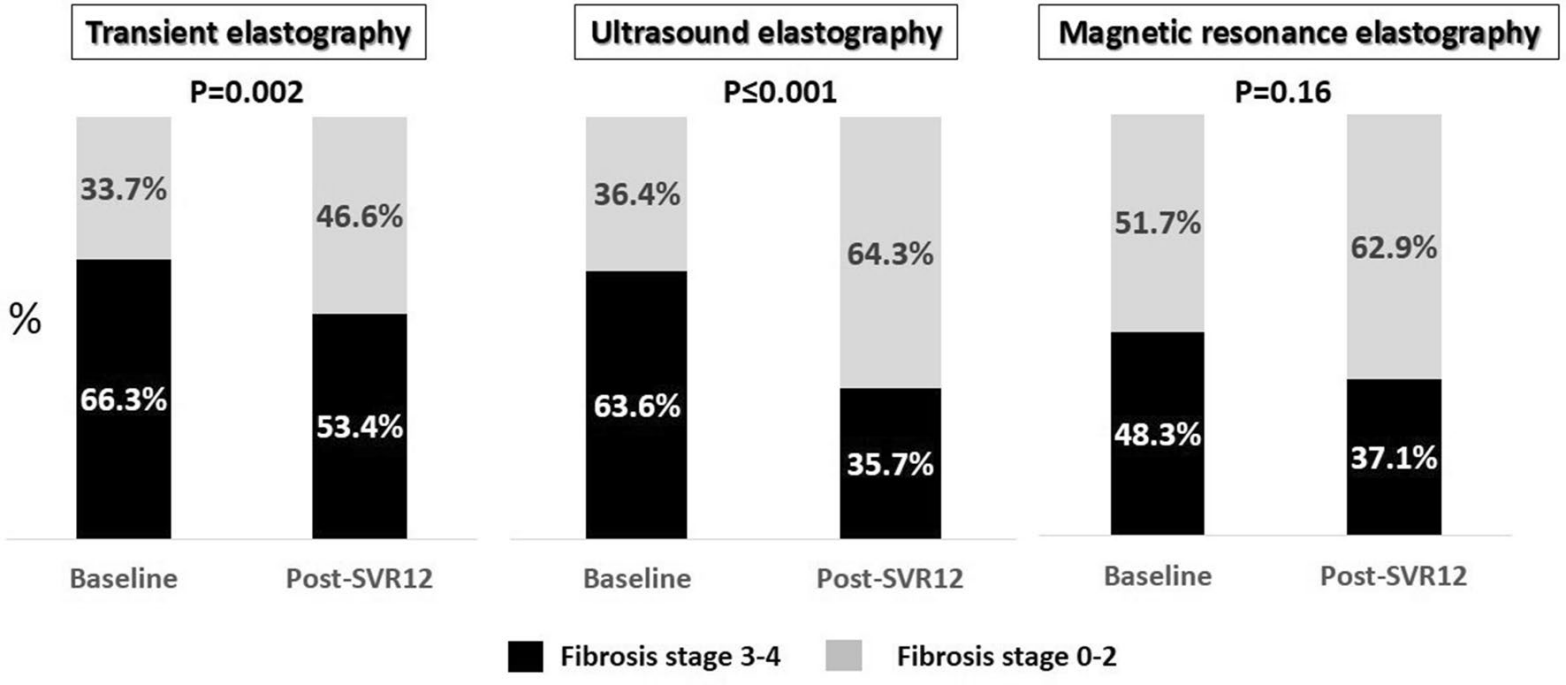
Proportion of advanced liver fibrosis evaluated by TE, SWE and MRE before and one-year after treatment initiation in chronic HCV patients with SVR.

LS values assessed by TE (18.0 ± 14.2 vs. 12.7 ± 10.7 kPa, p < 0.001); SWE (10.5 ± 3.5 vs. 8.9 ± 3.6 kPa, p < 0.001); and MRE (4.3 ± 1.8 vs. 3.8 ± 2.0 kPa, p < 0.001) as well as APRI (1.5 ± 1.7 vs. 0.7 ± 0.6, p < 0.001); and FIB-4 score (3.7 ± 3.1 vs. 2.8 ± 2.0, p < 0.001) significantly decreased from baseline to post-SVR12 (Fig. 4). The percentage of patients with advanced liver fibrosis (≥ stage 3 fibrosis) at post-SVR12 was notably reduced compared to baseline when assessed by TE (66.3% vs. 53.4%, p = 0.002) and SWE (63.6% vs. 35.7%, p ≤ 0.001) while it was not significantly decreased as assessed by MRE (48.3% vs. 37.1%, p = 0.16) (Fig. 5). Viral eradication resulted in the reduction of LS values as evaluated by three elastography techniques. Liver steatosis as evaluated by TE-based CAP (220.9 ± 49.9 vs. 234.0 ± 61.1 dB/m, p = 0.07) and MRI-determined PDFF (4.9 ± 3.6 vs. 3.8 ± 2.7%, p = 0.06) did not change from baseline to post-SVR12 (Supplementary Fig. 2).

### Clinical characteristics of non-obese vs. obese NAFLD patients and change of liver fibrosis from baseline to 1-year follow-up

In the subgroup of 93 patients with NAFLD, 30 patients (32.3%) were classified as having non-obese NAFLD with a BMI < 25 kg/m^2^. Compared to obese patients with NAFLD, non-obese patients had decreased waist circumference (34.1 ± 10.6 vs. 37.3 ± 4.1 inches, p < 0.001); WBC count (6.2 ± 1.7 vs. 7.5 ± 1.5 × 10^9^/L, p = 0.01); and ALT levels (34.9 ± 17.0 vs. 55.6 ± 49.6 IU/L, p = 0.03). Between non-obese and obese NAFLD patients, there was no significant difference in the prevalence of diabetes, haemoglobin, platelet count, TB, AST, albumin, fasting plasma glucose, HbA1C, and lipids. Furthermore, regarding the liver fibrosis and steatosis, non-obese patients with NAFLD had lower LS values as evaluated by TE (5.5 ± 2.0 vs. 7.4 ± 3.8 kPa, p = 0.01) and SWE (5.3 ± 1.6 vs. 6.2 ± 2.0 kPa, p = 0.03) as well as lower liver steatosis measured using TE-based CAP (242.5 ± 46.3 vs. 303.1 ± 51.8 dB/m, p < 0.001) and MRI-based PDFF (7.1 ± 6.4 vs. 13.3 ± 8.5%, p < 0.001) than obese patients with NAFLD. There was a trend toward lower LS as assessed using MRE (2.2 ± 0.5 vs. 2.4 ± 0.5 kPa, p = 0.07) in non-obese NAFLD patients than obese NAFLD patients. The APRI (0.3 ± 0.1 vs. 0.3 ± 0.3, p = 0.88) and FIB-4 scores (1.2 ± 0.7 vs. 0.9 ± 0.6, p = 0.06) were not different between obese and non-obese patients with NAFLD (Table 3).

**Table 3:** Baseline characteristics and change of serum simple fibrotic marker, elastography and steatosis value in Lean and obese NAFLD.

	Lean (n = 30)	Obese (n = 63)	p-value
**Baseline characteristics**			
Age (years)	55.7 ± 14.6	45.9 ± 12.6	0.003
Diabetes, n (%)	2 (7.1%)	13 (21.3%)	0.10
Body mass index (kg/m^2^)	22.7 ± 1.5	29.9 ± 5.1	< 0.001
Waist circumference (inches)	34.1 ± 10.6	37.3 ± 4.1	< 0.001
Hemoglobin (g/dL)	13.9 ± 1.3	13.9 ± 1.6	0.94
White blood cell (× 10^3^/μL)	6.2 ± 1.7	7.5 ± 1.5	0.01
Platelet count (× 10^3^/μL)	263.4 ± 58.5	277.8 ± 64.8	0.40
Total bilirubin (mg/dL)	0.6 ± 0.3	0.8 ± 0.4	0.24
AST (IU/L)	28.0 ± 12.1	33.3 ± 22.1	0.40
ALT (IU/L)	34.9 ± 17.0	55.6 ± 49.6	0.03
Albumin (g/dL)	4.3 ± 0.2	4.3 ± 0.3	0.78
FBS (mg/dL)	107.4 ± 15.0	110.0 ± 25.1	0.70
HbA1C (mg%)	5.6 ± 0.5	5.9 ± 0.9	0.20
Triglyceride (mg/dL)	134.7 ± 57.6	159.2 ± 64.0	0.13
LDL (mg/dL)	122.9 ± 37.1	129.2 ± 43.6	0.55
Cholesterol (mg/dL)	193.4 ± 51.0	203.4 ± 54.9	0.40
HDL (mg/dL)	48.8 ± 15.2	52.3 ± 27.9	0.98
APRI score	0.3 ± 0.1	0.3 ± 0.3	0.88
FIB-4 score	1.2 ± 0.7	0.9 ± 0.6	0.06
Transient elastography (kilopascal)	5.5 ± 2.0	7.4 ± 3.8	0.01
SWE (kilopascal)	5.3 ± 1.6	6.2 ± 2.0	0.03
MRE (kilopascal)	2.2 ± 0.5	2.4 ± 0.5	0.07
CAP (dB/m)	242.5 ± 46.3	303.1 ± 51.8	<0.001
MRI-PDFF (%)	7.1 ± 6.4	13.3 ± 8.5	<0.001
**At 1-year follow-up**			
Fibrosis progression, n (%)	2 (6.7%)	6 (9.5%)	0.45
BWBL-BW1Y (kg)	− 0.6 ± 2.3	0.6 ± 5.1	0.21
ALTBL-ALT1Y (IU/L)	3.0 ± 19.0	5.6 ± 34.0	0.81
TEBL-TE1Y (kilopascal)	0.5 ± 1.4	− 0.3 ± 2.4	0.15
SWEBL-SWE1Y (kilopascal)	0.5 ± 1.3	0.5 ± 1.8	0.65
MREBL-MRE1Y (kilopascal)	0.3 ± 0.7	− 0.1 ± 0.8	0.02
CAPBL-CAP1Y (dB/m)	− 15.2 ± 42.3	27.7 ± 90.6	0.01
MRI-PDFFBL-MRI-PDFF1Y (%)	− 0.8 ± 4.2	0.1 ± 5.5	0.78

*AST* aspartate aminotransferase, *ALT* alanine aminotransferase, *APRI* AST to Platelet Ratio, *BW* body weight, *BL* baseline, *CAP* controlled attenuation parameter, *FBS* fasting blood sugar, *FIB-4* Fibrosis-4, *HbA1c* Glycated haemoglobin, *HDL* high density lipoprotein, *LDL* low density lipoprotein, *MRE* magnetic resonance elastography, *MRI* magnetic resonance imaging, *PDFF* proton density fat fraction, *SWE* shear wave elastography, *TE* transient elastography.

At the 1-year follow-up, there was no significant change from baseline with respect to body weight, APRI, FIB-4 index, LS values as evaluated using imaging tests, liver steatosis values assessed by TE-based CAP, and MRI-PDFF in both obese and non-obese patients with NAFLD. Besides, the proportions of patients who had liver fibrosis progression, defined as an increase by at least one stage of fibrosis evaluated by MRE from baseline to the 1-year follow-up, was not different between patients with non-obese and obese NAFLD (6.7% vs. 9.5%, p = 0.45). Table 3 shows a comparison of the mean changes of simple serum fibrotic score, LS, and steatosis values at baseline and the 1-year follow-up in obese and non-obese patients with NAFLD.

## Discussion

The current study investigated the correlation of simple serum fibrosis score, LS, and liver steatosis values evaluated by imaging methods in two groups of patients with CLDs, including chronic HCV infection and NAFLD, and then tracked changes in these values over a year. To our knowledge, this is the first study to simultaneously use the three currently available elastography tests to evaluate LS. Our main findings are: (i) There are significantly strong positive correlations of LS values assessed among the three elastography techniques. At the same time, there is a moderate-to-strong correlation between imaging methods and non-invasive serum fibrosis scores. Liver steatosis evaluated by TE-based CAP is moderately correlated with MRI-PDFF. (ii) Using MRE as the reference method for the staging of liver fibrosis, TE, SWE, APRI, and FIB-4 index were accurate in diagnosing significant fibrosis (≥ F2), advanced fibrosis (≥ F3), and cirrhosis (F4). (iii) Viral eradication after DAAs therapy in chronic HCV patients improves APRI, FIB-4 score, and LS as assessed by TE, SWE, and MRE. In contrast, liver steatosis measured by TE-based CAP and MRI-PDFF does not change after SVR. (iv) Non-obese patients with NAFLD have lower LS and steatosis values than obese patients with NAFLD. LS and steatosis did not change after 1 year of follow-up in non-obese and obese patients with NAFLD.

Non-invasive tests for assessment of liver fibrosis are becoming more desirable and widely used to improve the diagnosis and prognostication of CLD of various aetiologies. TE, SWE, and MRE exhibited excellent diagnostic accuracy for detecting liver fibrosis and have emerged into clinical practice and research purposes^22,23^. However, data on the correlations among three elastography techniques in patients with CLD patients are scarce. Our study showed a good correlation among imaging elastography tests and between imaging tests and simple serum biomarkers, consistent with a previous study in which MRE and TE showed moderate correlation^24^. MRE can be considered the most accurate NIT for detecting liver fibrosis, especially in advanced fibrosis and cirrhosis^25^. In a recent study, MRE outperformed TE and SWE in detecting stage 4 liver fibrosis in patients with NAFLD^22^. Furthermore, MRE has an advantage over TE or SWE in that it visualizes the whole liver rather than just small hepatic areas. Hence, MRE was selected as the reference method for the staging of liver fibrosis in the current study. Overall, TE, SWE, APRI, and FIB-4 scores had a high diagnostic performance for detecting stage 1–4 liver fibrosis. The accuracy of all tests was comparable. Thus, although MRE is currently accepted as the most precise NIT for evaluating liver fibrosis, our study showed that TE, SWE, APRI, and FIB-4 had a good correlation and can be used particularly in centres with limited availability of MRE. In the current study, imaging findings revealed that 45 out of 182 patients had cirrhosis. Of these, 29 patients (64.4%) had MRE-based LS cut-off ≥ 4.6 kPa (F4). The discrepancy between MRE and radiological results might be explained by interobserver variability among radiologists.

Liver necroinflammation influences the increase in LS as evaluated by imaging-based elastography^26–28^. Therefore, the presence of advanced fibrosis or cirrhosis assessed by LS measurement in patients with chronic HCV prior to DAA treatment might be overestimated. Previous studies used TE^29,30^ or MRE^31^ to show that the LS values reduced from baseline to SVR24 in patients with chronic HCV treated with DAA. Our study demonstrated that the LS value based on three elastography tests, APRI, and FIB-4 index in chronic HCV patients with SVR significantly decreased from baseline to 1 year following DAA treatment. Viral eradication resulted in a reduced proportion of patients with advanced fibrosis by 13%, 28%, and 11% based on TE, SWE, and MRE, respectively. Elevated ALT levels, which indicate the presence of necroinflammatory activity, decreased between baseline and SVR12 and between SVR12 and the 1-year follow-up. Thus, the reduction of LS values and serum fibrosis scores after viral eradication were partly caused by the decrease in liver inflammation rather than liver fibrosis regression. Another previous study showed that the improvement of liver fibrosis was not evident in 20 paired biopsies in the short-term of chronic HCV patients who achieved SVR after DAA treatment^32^. Reassessing LS after achieving SVR might be more accurate for non-invasive diagnosis of advanced fibrosis and be helpful information for clinical decision-making on patient follow-up. Moreover, liver steatosis values based on Fibroscan-based CAP and MRI-PDFF were not different between the baseline and 1-year follow-up after initiation of DAA therapy in patients with chronic HCV. This was inconsistent with Tada et al. study, which showed that viral eradication improved MRI-PDFF values at SVR24^31^. The possible reason for the discordant results between both studies may be the difference of HCV genotype (47.4% genotype 1b/52.6% genotype 2 vs. 46.0% genotype 1/44.9% genotype 3) and time of repeated liver steatosis measurement (24 weeks after end-of-treatment vs. 24–36 weeks after end-of-treatment) between Tada et al. study and our study^31^. HCV genotype is strongly associated with the prevalence of liver steatosis. Genotype 3 infection is now commonly recognized as an independent aetiological factor of steatosis^33,34^.

The prevalence of lean NAFLD varies from 5–26%, although it ranges from 5–45% among Asians and 5–20% in European populations^35,36^. It is unclear whether the clinical and histological characteristics and prognosis differ between obese and non-obese patients with NAFLD. A recent study by Younes et al. showed that despite the fact that non-obese patients with NAFLD patients had less severe histological disease, advanced fibrosis, and lower prevalence of diabetes, they still experienced similar long-term prognosis and survival compared to obese patients with NAFLD^37^. A prospective Chinese cohort of biopsy-proven NAFLD patients showed that non-obese NAFLD patients had less metabolic syndrome, NAFLD activity score, and liver fibrosis and likely a better prognosis than obese patients^38^. In contrast, a European study found that even though non-obese patients with NAFLD had fewer metabolic syndrome components, they had severe histological features similar to obese patients and had more progressive disease than obese NAFLD patients^39^. Data on liver stiffness and steatosis evaluated by imaging methods in non-obese compared to obese NAFLD patients are limited. The current study demonstrated that non-obese patients with NAFLD had less LS and liver steatosis, while they had a similar metabolic profile with similar dyslipidaemia and glycaemia when compared to patient with obese NAFLD. Changes in BMI, serum fibrosis score, LS, and steatosis values were not different between obese and non-obese patients with NAFLD at the 1-year follow-up. The reason for different observations among these studies may be explained by a major confounding factor of the younger age of non-obese patients, which has been mentioned in previous studies^37,40^. However, it is possible that this is not the true reason given that the mean age of non-obese and obese NAFLD patients was not significantly different in the Chinese and European studies. Additionally, non-obese patients in our study were older than obese patients. Further studies are needed for the long-term progression of LS and steatosis evaluated by NITs and to compare both groups of NAFLD patients.

This study has some limitations. First, the number of patients and the period of follow-up were both relatively small. Further large-scale studies with longer follow-up are needed to confirm these findings. Second, the study lacked liver biopsy results. Despite several limitations of liver biopsies, the validation process using biopsy is still mandatory to evaluate the performance of a new test. However, the performance of several tests can be assessed without the need for biopsy. Furthermore, TE, SWE, and MRE have been extensively validated in a large number of studies. Third, all TE, SWE, and MRE evaluations were performed on the same equipment for each technique. Therefore, extrapolating our findings to the clinical setting with various MRI or ultrasound models might not be possible. Fourth, the number of patients in each fibrosis stage was unequal, with 70, 42, 26, 7, and 37 patients having F0, F1, F2, F3, and F4, respectively. However, this might not have a significant impact on the results since the primary objective of the study was to assess the diagnostic performance of current NITs value in patients with chronic liver diseases. Fifth, despite the fact that hepatic inflammation affects the accuracy of NITs for liver fibrosis staging, patients with elevated aminotransferase were not excluded from studying the change of NITs value after HCV eradication. Moreover, only 8/182 (4.4%) patients had baseline serum ALT levels greater than five times the upper limit of normal.

In conclusion, TE, SWE, MRE, APRI, and the FIB-4 index have a good correlation. Using MRE as the reference standard, all studied NITs have high diagnostic accuracies for detecting liver fibrosis. Viral eradication after DAA therapy in chronic HCV patients results in the improvement of APRI, FIB-4 score, and LS as assessed by TE, SWE, and MRE. Non-obese NAFLD patients have lesser LS and steatosis than obese NAFLD patients, but changing of LS and steatosis are not different after short-term follow-up.

## Supplementary Information

Supplementary Information.

## Data Availability

The data that support the findings of this study are available from the corresponding author upon reasonable request.
