# Supplementary material for: Non-invasive tests for liver fibrosis assessment in patients with chronic liver diseases: a prospective study
Source: Sci Rep. 2022 Mar 22;12:4913. doi: 10.1038/s41598-022-08955-x (PMC8941081; doi:10.1038/s41598-022-08955-x)
Supplement: Supplementary file 1 — Supplementary Information. [file 41598_2022_8955_MOESM1_ESM.docx]

**Supplementary figure 1: Distribution of the LS values by TE (a) and SWE (b) in comparison with different fibrosis stages using MRE**

**
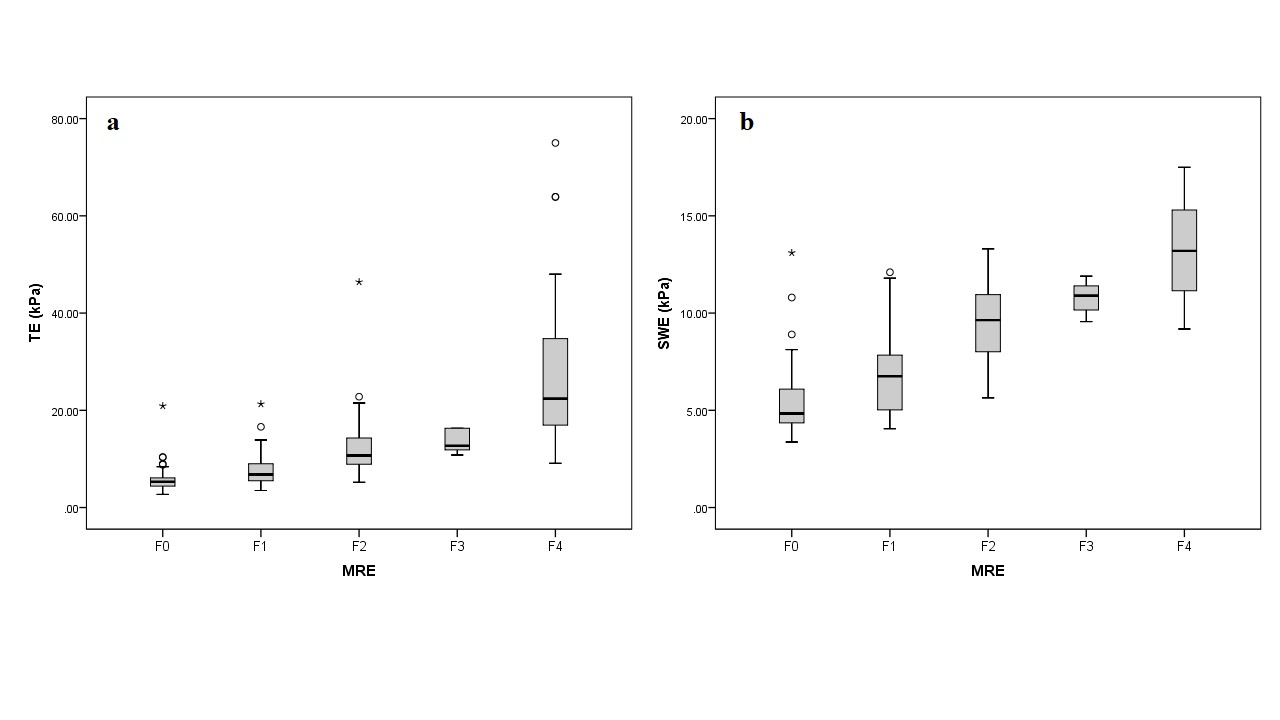
**

**Supplementary figure 2: TE-based CAP and MRI-PDFF at baseline and post-SVR 12 in chronic HCV patients with SVR after DAA treatment**

**
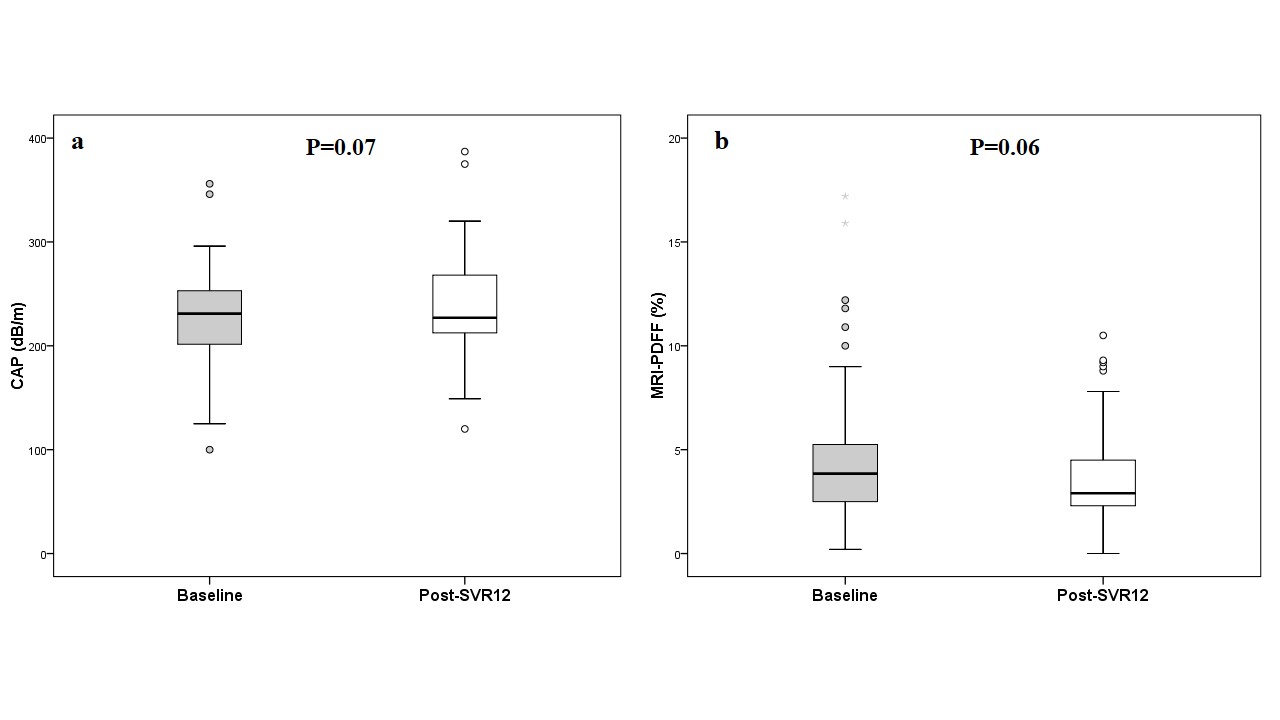
**

**Supplementary table 1: The average of TE, SWE, APRI and FIB-4 score according to fibrosis stage using MRE as reference method**

| **Mean ± SD** | **F0 (n=70)** | **F1 (n=42)** | **F2 (n=26)** | **F3 (n=7)** | **F4 (n=37)** |
| --- | --- | --- | --- | --- | --- |
| TE, kPa | 5.7 ± 2.4 | 7.8 ± 3.6 | 13.3 ± 8.3 | 13.7 ± 2.5 | 28.0 ± 15.7 |
| SWE, kPa | 5.4 ± 1.7 | 6.7 ± 1.9 | 9.4 ± 2.1 | 10.8 ± 0.9 | 13.2 ± 2.4 |
| APRI score | 0.30 ± 0.22 | 0.48 ± 0.32 | 1.00 ± 0.77 | 1.29 ± 1.11 | 2.35 ± 2.10 |
| FIB-4 score | 1.04 ± 0.86 | 1.35 ± 0.85 | 2.18 ± 1.28 | 3.75 ± 2.47 | 5.53 ± 3.73 |
